# Acknowledgement to Reviewers of *Marine Drugs* in 2013

**DOI:** 10.3390/md12021160

**Published:** 2014-02-24

**Authors:** Marine Drugs Editorial Office

**Affiliations:** MDPI AG, Klybeckstrasse 64, CH-4057 Basel, Switzerland

The editors of *Marine Drugs* would like to express their sincere gratitude to the following reviewers for assessing manuscripts in 2013: 

Abdelmohsen, Usama RamadanArias, HugoBates, StephenAbreu, PedroArias, Maria CeciliaBattaglia, LuigiAdachi, MasaoArkhipova, Irina R.Baughn, Anthony D.Adams, NickAsai, TeigoBecker, PierreAfiyatullov, Shamil Sh.Asakawa, YoshinoriBedini, EmilianoAgbaga, Martin-PaulAytekin, A. OzhanBellei, BarbaraAgyei, DominicAyyalusamy, RamamoorthyBeniash, E.Aitken, Robert JohnAzadi, ParastooBenkendorff, KirstenAkk, GustavAzam, LaylaBen-Shabat, ShimonAlfonso, AmparoAzuma, KazuoBerdyshev, EvgenyAlloatti, GiuseppeBagno, AlessandroBeretta, GiovanniAlonso, AlejandraBaker, BillBerger, WalterAlvarez, Ana I.Baldwin, Susan A.Bergès, ThierryAlves, AnabelaBalskus, Emily P.Bero, JoanneAmade, PhilippeBaltz, Richard H.Berry, John P.Amy, PennyBalunas, Marcy J.Berta, Giovanni NicolaoAnderson, Clarissa R.Bandarra, Narcisa MariaBiagini, GiuseppeAndjelkovic, Anuska V.Banerjee, DebabrataBickmeyer, UlfAndolfi, AnnaBanerjee, MonimoyBilan, MariaAndrade, Paula BranquinhoBankova, VassyaBisignano, GiuseppeAndrianasolo, EricBanoub, Joseph H.Blanchfield, JoanneAngeles, Thelma S.Banskota, Arjun H.Blázquez, MaríaAnraku, MakotoBaptista, Mafalda S.Blunt, John W.Apáti, ÁgotaBarbosa, Teresa M.Bøgwald, JarlArai, MasayoshiBargu, SibelBohlin, LarsArbogast, SandrineBasti, LeilaBondon, ArnaudBongarzone, SalvatoreCarrano, LuciaClark, RichardBonheyo, GeorgeCarter, DarrickClericuzio, MarcoBontemps, NatalyCasapullo, AgostinoCodd, RachelBorchman, DouglasCasely-Hayford, MaxwellCollado, Isidro G.Bosmans, FrankCastell, AlinaConcannon, CaoimhinBotana, LuisCastro, AnaCondezo-Hoyos, L.Bourdelais, AndreaCavalieri, VincenzoCooksey, KeithBourdon, J.A.Cavalli, RobertaCooper, Edwin L.Bowden, BruceCavanagh, JohnCooper, William T.Boyle, Joseph J.Celik-Ozenci, CilerCordoba-Diaz, D.Braig, SimoneCeron, JoseCorsaro, Maria MichelaBrase, StefanChabert, PhilippeCorte-Real, ManuelaBrash, A.R.Chai, Sergio C.Costa-Lotufo, Letícia V.Breitling, RainerChakrabarti, DebopamCostantini, MariaBrett, Michael T.Chang, Fang-RongCostantino, ValeriaBright, J.J.Chang, Wen-ChengCosta-Rodrigues, JoãoBrinkman, Diane L.Châtel, AmélieCoyne, KathrynBroggini, MassimoChen, BingCresteil, ThierryBruhn, TorstenChen, Jih-JungCretoiu, Mariana SilviaBrunet, ChristopheChen, Jin-JerCristina, TeixidóBruno, LauraChen, QianCudic, MareBruno, M.Chen, Qi-YinCurnow, PaulBüttner, SabrinaChen, ShawnCusick, Kathleen D.Bugni, Tim S.Cheng, Shi-YieD’abrosca, BrigidaBuisson, DidierCheng, ShuD’ursi, PasqualinaBurgos, Javier S.Cheng, Yuan-BinD’Acquisto, FulvioButler, MarkCherian, PhilipDale, BrianButler, Mark S.Chew, BoonDaly, NorelleButts, Craig P.Choi, Hong SeokDamodaran, Vinod BabuBykov, Vladimir J.N.Choi, Jong-ilDangardt, FridaCabado, Ana G.Choi, Yung HyunDattelbaum, Jonathan D.Cabot, MylesChooi, Yit-HengDavenport, Ian R.Cabrita, Maria TeresaChristaki, EfterpiDe Agostini, ArianeCalado, RicardoChristie-Oleza, JosephDe Castro, CristinaCalder, PhilipChung, ChuhanDe Felício, RafaelCaldwell, GaryChung, Hun-TaegDe Koning, Harry P.Caldwell, Kim A.Church, W. BretDe La Vega, Ricardo R.Cammarata, MatteoCianca, Rosa C. CervantesDe Lera, Angel R.Canki, MarioCiapetti, GabrielaDe Marino, SimonaCapper, AngelaCiardelli, GianlucaDe Mendonça, Dina I.M.D.Cardoso, Susana M.Ciavatta, Maria LetiziaDe Morais, Rui Manuel Santos CostaCarmeli, ShmuelCiufolini, MarcoDe Pascale, DonatellaEnomoto, KeiichFrohbergh, MichaelDe Riccardis, FrancescoEriksson, MatsFroscio, Ssuzanne M.De Rosa, SalvatoreEspiña, BegoñaFuentes-Grünewald, C.De Troch, MarleenEsteves, Ana I.S.Fuwa, HaruhikoDeeds, Jonathan R.Evans, Bradley S.Gaboury, LouisDelfourne, EvelyneEwart, H. StephenGademann, KarlDemadis, KostasFahmy, Hesham T.Y.Galano, Jean-MarieDesaubry, LaurentFalconer, Ian R.Gallo, AlessandraDesideri, AlessandroFalkowski, PaulGalluzzi, L.Devenish, RodFalson, PierreGandour, RichardDiana, PatriziaFaraoni, DavidGanesan, A.Díaz-Marrero, Ana R.Farrell, HazelGarcia-Cuetos, LydiaDickschat, Jeroen S.Fassouane, AzizGarner, AmandaDimas, KonstantinosFaulkner, SimonGazdar, AdiDiogene, JorgeFecik, RobertGbogouri, AlbarinDittmann, ElkeFedorenko, VictorGelman, FainaDoerrler, WilliamFeng, RentianGenilloud, OlgaDomínguez, ElenaFérézou, Jean-PierreGenovese, GiuseppaDomínguez, HerminiaFernández, José J.George, Sarah J.Dong, JixinFewer, DavidGetchell, RodmanDooley, HelenFiebig, Heinz HerbertGhayur, Muhammad NabeelDornetshuber, RitaFigueroa, Félix LópezGilmour, JimDoroghazi, James R.Fisher, Aron B.Glaser, Keith B.Døskeland, SteinFitton, Janet HelenGlencross, BrettDowning, TimFleming, LoraGochfeld, DeborahDreanno, CatherineFlorence, Gordon J.Goh, Beverly Pi LeeDrewry, David H.Florent, IsabelleGomez-Escribano, Juan-PabloDuarte, NoéliaFlorio, TullioGordaliza, M.Duh, Chang-YihFlower, DarrenGottesman, Michael M.Durkin, ColleenForano, EvelyneGrabarczyk, MałgorzataDurnford, DionForim, Moacir RossiGram, LoneDwivedi, ChandradharForzato, CristinaGrassi, GiovanniEberhart, Bich-Thuy L.Foster, Michelle T.Gray, Christopher A.Edison, Arthur S.Frame, ElizabethGreen, David W.Edrada-Ebel, RuAngelieFranco, ChrisGregory, Melissa K.Edwards, AndreaFranco, José M.Grenha, AnaEgan, SuhelenFranco, OctavioGrkovic, TanjaEirín-López, José M.Frank, UriGrogan, GideonElsebai, Mahmoud FahmiFranzetti, AndreaGrovel, OlivierElsinghorst, Paul W.Freeman, Christopher J.Gugger, MurielEly, B.Freitas, Ana C.Gugliandolo, ConcettaEmblem, ÅseFrisvad, Jens ChristianGulder, TobiasGupta, PrasoonHolmes, PhilipJiménez, CarlosGupta, Subash C.Holst, OttoJin, ZhendongGuschina, Irina A.Honoré Hansen, SteenJoo, Hong-GuGustafson, KirkHopkins, AnnJos, A.Guzmán, Esther A.Hoshi, NaotoKaasalainen, UllaHa, Ki-TaeHosokawa, MasashiKalaitzis, JohnHäder, Donat-P.Hou, GuangjinKalimuthu, SenthilkumarHaft, Daniel H.Hou, YanpengKalinin, VladimirHagenbuch, BrunoHowie, SarahKan, ToshiyukiHajduch, EricHu, YoucaiKaneko, JunHallegraeff, Gustaaf M.Huang, Dorothy YuKashman, YoelHallsworth, J.E.Huang, ShengxiongKaufmann, KatrinHanif, NovriyandiHuang, Shi-YingKawai, ShigeyukiHansen, EspenHuet, EricKawakami, KojiHanski, LeenaHughes, Francis M.Kawasaki, TomomiHarder, TilmannHuigens III, Robert W.Kearns, Philip S.Harinantenaina, LivaHungerford, JamesKeatinge-Clay, AdrianHarnedy, PádraigínHunter, ChristyKellenberger, EstherHashidoko, YasuyukiHwang, Tsong-LongKellenberger, StephanHawinkels, Lukas J.A.C.Ibanez, ElenaKelley, Darshan S.Hayashi, MasahiroImhoff, JohannesKellmann, RalfHayen, HeikoIoannou, EfstathiaKelly, WendyHelbert, WilliamIrhimeh, Mohammad R.Kem, WilliamHellmich, ChristianIshibashi, MasamiKenttämaa, Hilkka I.Helm, Richard F.Ishiki, ManabuKerr, SteveHelms, VolkhardIskandarov, UmidjonKhadem, ShahriarHenrich, Curtis J.Ito, TakeoKhanna, RajeshHenriques, SoniaIto, TatsuyaKhozin-Goldberg, InnaHeo, Soo-JinItoi, S.Kiene, Ronald P.Heretsch, PhilippIwagawa, TetsuoKikuchi, HaruhisaHerfindal, LarsIwao, MasatomoKilcoyne, JaneHernández-Losa, JavierJachymek, WojciechKim, Gwang HoonHerr, IngridJacobson, PeerKim, Hye KyongHerraiz, TomásJansen, RolfKim, Ji HoeHerrero, M.Jeannerat, DamienKim, PaulHidari, Kazuya I.P.J.Jégou, CamilleKim, Se-KwonHildebrand, MarkJeon, You-JinKim, Sung-HoonHintze, VeraJesus Raposo, Maria FilomenaKinnear, SusanHirokawa, TakatsuguJiang, Lin-HuaKinsey, William H.Hoeger, UlrichJiang, SunnyKishimoto, YoshimiHohensinner, PhilippJiao, GuanglingKita, YasuyukiHolland, PatrickJiménez-Barbero, JesúsKletsas, DimitrisKlint, JulieLauder, BobLurling, MiquelKluza, JeromeLaurienzo, PaolaLutz, RichardKnölker, Hans-JoachimLauritano, ChiaraMa, Edmond Dik-LungKobayashi, MakitoLay, Horng-LiangMacMillan, John B.Kobayashi, YasuhisaLaycock, Maurice V.Madeo, FrankKöcher, ThomasLeal, MiguelMadhukar, BurraKodani, ShinyaLebeau, ThierryMaeda, HayatoKolaczkowski, MarcinLeblond, JeffreyMaes, LouisKomoroski, Richard A.Lee, Sang KookMaier, Jeanette A.M.Kong, FangmingLee, Seung-MinMaisch, TimKonoki, KeiichiLee, Wen-SenMajik, MaheshKornprobst, Jean MichelLefay, CatherineMalgesini, BeatriceKorsnes, Mónica SuárezLehotay, Steven J.Maloney, KatherineKoshino, HiroyukiLehrer, Robert I.Mamoun, Choukri BenKoskinen, Ari M.P.Leikoski, NiinaMancini, InesKotoku, NaoyukiLeone, AntonellaMándi, AttilaKourist, RobertLesueur, CelineManfrin, ChiaraKovbasnjuk, OlgaLewandowski, PaMangoni, AlfonsoKraan, StefanLi, HuabinMånsson, MariaKrepkiy, Dmitriy V.Li, KeManzo, EmilianoKrock, BerndLi, Lu-PingMao, Simon J.T.Krüger, ThomasLiermann, Johannes C.Marchetti, PhilippeKubo, YoshinaoLim, Yun-PingMarchioli, RobertoKubota, TakaakiLin, ZhichengMarco-Contelles, JoséKuczer, MariolaLinder, StefanMariottini, Gian LuigiKujawinski, Elizabeth B.Lindholm, DanMarkopoulos, JohnKumirska, JolantaLiras, PalomaMarron, Alan O.Kuo, Jen-MinLitaker, WayneMartinez, RonnyKuroda, ChiakiLittlefield, Bruce A.Martínez-Banaclocha, Marcos ArturoKurosawa, NorioLiu, DelongKurtboke, IpekLiu, JinMartinez, AnaKwon, Young M.Llewellyn, CaroleMásson, MárLai, DaowanLong, Benedict M.Mastinu, AndreaLaird, DamianLongstaff, ColinMata, LeonardoLamarche, Matthew J.Lönnberg, HarriMatsuda, FumioLampert, AngelikaLopanik, Nicole B.Matsunaga, ShigekiLandini, PaoloLopata, Andreas L.Matsuya, YujiLane, Amy L.Lopez-Huertas, EduardoMatthews, Jacqueline M.Lang, SiegmundLordan, SinéadMazurak, VeraLanzetta, RosaLu, Chung-KuangMazzei, PierluigiLatortue, Marie C.Lu, JunMcAlpine, ShelliLau-Cam, Cesar A.Lundholm, NinaMcarthur, JeffMccarthy, PeterMunday, RexOkamoto, SusumuMcCarty, MarkMuramoto, KojiOkamoto, YoshiharuMcdougal, Owen M.Murphy, KarenOkello, EdwardMcgregor, Glenn B.Murphy, Patrick J.Olafsdottir, Elin SoffiaMckee, Lauren S.Muylaert, KoenraadOliva, M.Mclean, Timothy I.Nacke, HeikoOliveira Rocha, Hugo AlexandreMcmanus, Owen B.Nagai, HiroshiOlsen, ChristianMebs, DietrichNair, Satish K.Omarsdottir, SesseljaMedina, Miguel A’ngelNaka, HiroakiOntsouka, Edgar CorneilleMehiri, MohamedNakao, YoichiOpatz, TillMenna, MarialuisaNapier, JohnathanOrtíz, Abel BaergaMi, Fwu-LongNappo, MichelaOsaki, TomohiroMitchell, JohnNavacchia, Maria LuisaOstenfeld Larsen, ThomasMiyaoka, HiroakiNestor, CarballeiraÖstman, ÖrjanMizuno, MasashiNeuzil, JiriOuahid, YounessMoczydlowski, Edward G.Newman, DavidOvenden, SimonMoffit, MichelleNguyen, Anh-ThoOvissipour, MahmoudrezaMolgo, JordiNicoletti, RosarioPagliara, PatriziaMolinski, TadeuszNielsen, Thorbjørn TerndrupPahwa, SavitaMollica, AdrianoNijland, ReindertPallela, RamjeeMolnár, IstvánNiki, HironoriPalomares, Eva RufinoMontero, OlimpioNissen-Meyer, JonPalumbo, AnnaMontresor, MarinaNobili, StefaniaPark, Joo-InMonzavi-Karbassi, BehjatolahNoguchi, TamaoPark, WansuMoody, A. JohnNordon, Robert E.Parrington, JohnMoody, Christopher J.Norte, ManuelPassarelli, MarisaMoody, Terry W.Norte, MunuelPawel, PawelMoore-Kucera, JenniferNorthen, TrentPayo, Dioli AnnMorabito, MarinaNovelli, AntonelloPearce, A. NorrieMoran, YehuNumata, KeijiPei, Jin-JingMoreira, SusanaOberlies, Nicholas H.Pelkonen, OlaviMoreno, AndrésOberthür, MarkusPelletier, JerryMori, KenjiOchi, KozoPemberton, John M.Mori, NaokiOda, TatsuyaPeng, HanjingMorikawa, ToshioO’doherty, GeorgePeng, Jiang-NanMorimura, ShigeruOh, Dong-ChanPenna, AntonellaMorton, Steve L.Ohta, ShinjiPereira Neto, Ana MariaMorzycki, Jacek W.Oishi, ShinyaPereira, LeonelMoscow, Jeffrey A.Ojida, AkioPérez, Andy J.Motti, CherieOjika, MakotoPérez-Victoria, IgnacioMoussa, FathiOk, SalimPerhar, GurbirMüller, Werner E.G.Okada, SeijiPerrone, DanielaPescitelli, GennaroRialland, MickaëlSchenkel, JohannesPestov, A.V.Ribalet, FrancoisSchmid, AndreasPetrie, JamesRiccio, RaffaeleSchmidt, BorisPfeifer, GerdRice, ScottSchmidt, MarianePflugmacher, StephanRienzi, Sara DiSchmitz, RuthPicot, LaurentRobertson, Charles R.Schneider, ClausPiel, JörnRobinson, MarkSchock, TraceyPietro, A. DiRocchetti, MarcellaScholz, BettinaPiggott, AndrewRocha-Santos, Teresa A.P.Schröder, Heinz C.Piorko, AdamRodrigues, AliceSchuhmann, HolgerPires, O.R.Rodriguez, CarmenSchuller, Kathryn A.Pistocchi, RossellaRodríguez, Ileana I.Schulz, Joseph R.Pitsinos, Emmanuel N.Rodriguez, JaimeSchunck, Wolf-HagenPlouguerné, ErwanRohan, Lisa CenciaSchuster, DanielaPodechard, NormandRohrlack, ThomasSchwindt, AdamPodlasek, Carol A.Romano, GiovannaSeabra, VítorPohnert, GeorgRomerosa, AntonioSeca, AnaPomati, FrancescoRottinger, EricSégui, BrunoPomin, Vitor HugoRourke, WadeSeifert, KarlheinzPottinger, Tom G.Rousseau, MartheSellner, KevinPouchus, Yves FrançoisRoussis, VassiliosSeo, Jeong-WooPrêcheur, IsabelleRovero, PaoloSérgio, AlbuquerquePrescott, MarkRusso, Gian LuigiSerive, BenoitProksch, PeterRustad, TuridSewald, NorbertProteau, Philip J.Saito, NaokiSeymour, Justin R.Quesada, Ana R.Sakamoto, ToshioShaaban, MohamedQuesada, AntónioSalierno, James D.Sharma, SushilQuilliam, Michael A.Salomon, ChristineShea, TomQuinton, LoïcSamaras, PetrosSherman, DavidRadwan, Mohamed M.Sammut, Ivan A.Sheu, Jyh-HorngRaine, RobinSánchez-Moreno, ManuelShin-ya, KazuoRamis, JoanaSandjo, Louis PergaudSieber, JonasRamón Y. Cajal, SantiagoSantiago-Vazquez, LorySiegel, DionicioRasapalli, SirappaSaravanan, PalaniappanSieiro, CarmenRateb, Mostafa E.Sarmento, HugoSikorska, HannaRawson, AshishSass, HenrikSilipo, AlbaReft, AbigailSasso, SeverinSilva, LuísRein, KathleenSatin, JonathanSilva, Tiago H.Reitzel, AdamSatomi, YoshikoSimmet, ThomasRemias, DanielSavvidis, ΥiannisSimpson, Thomas J.Reyes, FernandoSayanova, OlgaSims, JamesRho, Jung-RaeScharf, Daniel H.Sinclair, AlexSinclair, AndrewSvenson, JohanTosi, SolveigSingh, RajeshSydnes, Magne O.Tosti, ElisabettaSire, OlivierSzentmiklosi, JozsefTricone, AntonioSlattery, MarcTaglialatela-Scafati, OrazioTrischman, JackieSmith, G. JasonTakahashi, KoretaroTrosko, James E.Smith, Renee J.Takaichi, ShinichiTrottein, FrançoisSmith, Terry K.Takemori, HiroshiTsuda, MasashiSmith-Palmer, TruisTaketani, Rodrigo GouvêaTurco, GianlucaSmyth, ThomasTanaka, Jun’ichiTwiner, Michael J.Sneed, JenniferTanaka, SachikoTytgat, JanSolecka, JolantaTanaka, TakujiUdenigwe, Chibuike C.Song, Peter I.Tang, Feng-YaoUeda, KatsuhiroSong, ZhihongTang, HongUhrin, DusanSoule, TanyaTantillo, DeanUlens, ChrisSouto, María L.Tapper, BrianUsami, YoshihideSpahn, ViolaTarantini, FrancescaUsov, Anatoly I.Spiteller, PeterTarapore, Rohinton S.Usuki, YoshinosukeSpoering, AmyTarman, KustiariyahUtkina, Natalia K.Srivastava, S.K.Tasker, AndrewValdiglesias, VanessaStaerk, DanTatituri, Raju Venkata VeeraVale, PauloStanstrup, JanTeas, JaneVan Mooy, Benjamin A.S.Staudinger, Jeff L.Teichert, Russell W.Van Pée, Karl-HeinzStensvag, KlaraTeijido, OscarVan Wagoner, Ryan M.Stern, RobertTejesvi, Mysore V.Varela, JoãoSteverding, DietmarTemeyer, Kevin B.Varotti, Fernando De PillaStierle, AndreaTeruya, KiichiroVarticovski, L.Stierle, DonaldTezuka, YasuhiroVarvounis, GeorgeStout, ElizabethThalhammer, TheresiaVasconcelos, VitorStrathmann, Frederick G.Thany, SteeveVelkov, TonySu, Jui-HsinTheodorakis, Emmanuel A.Venable, Mark E.Su, XiongThessen, AnneVenier, PaolaSubramani, RameshThiel, TeresaVennekens, RudiSuchana, AppleThiéry, ValérieVéron, BenoîtSuenaga, KiyotakeThomas, Olivier P.Vetvicka, VaclavSüssmuth, RoderichThomas, TorstenViaggiu, EmanuelaSugiyama, TetsuyaThomes, PaulVian, Maryline AbertSugiyama, TsuyoshiTobe, KerstinViana, Gustavo H.R.Sugumaran, ManickamToida, ToshihikoVicente, JanSumarah, MarkToma, LucioVidoudez, CharlesSung, Ping-JyunTomás, JuanVignisse, JulieSun-Ju, KimTominaga, AkiraVilariño, NataliaSuzuki, HodakaTomm, Janina M.Vinogradov, EvgenyVitetta, LuisWijesekara, IsuruZahler, StefanVon Elert, EricWilliams, DavidZähringer, UlrichVon Schacky, ClemensWilliams, Robert M.Zakarian, ArmenWade, John D.Wilson, Peter DZaki, Noha M.Wahlsten, MattiWinberg, PiaZampella, AngelaWakimoto, ToshiyukiWittlin, SergioZan, JindongWalsh, Catherine J.Witulski, BernhardZanchetta, PhilippeWalsh, DavidWolberg, Alisa S.Zeeck, AxelWang, ShuWörmer, LarsZengler, KarstenWang, TianfangWright, Christine E.Zervou, MariaWang, Wei-HsienWu, Shih-HsiungZhang, DahaiWang, Yan-HuaWu, Yang-ChangZhang, HongliangWarren, C. R.Yamashita, EijiZhang, JunzengWarren, J. DavidYan, AixinZhang, XunWatabe, ShugoYang, BooZhang, YongWatts, KatharineYang, Hsin-LingZheng, Qi-HuangWei, MeiYang, Yu-LiangZhou, EdwardWeiss, JohnYang, ZhicaiZhou, MeiWeißflog, JerritYarotskyy, ViktorZhou, Yu-DongWen, Zhi-HongYe, TaoZöller, MargotWertz, PhilipYih, Ling-HueiZorzano, AntonioWestermann, BernhardYoshida, HiroshiZotchev, SergeyWestwell, Andrew D.Yoshikawa, KazukoZubía, EvaWever, RonYotsu-Yamashita, MariWhite, Robert L.Yuan, TaoWickenden, AlanZachariae, Ulrich

